# Improved Analysis for Intrinsic Properties of Triaxial Accelerometers to Reduce Calibration Uncertainty

**DOI:** 10.3390/mi15121494

**Published:** 2024-12-14

**Authors:** Jon Geist, Hany Metry, Aldo Adrian Garcia Gonzalez, Arturo Ruiz Rueda, Giancarlo Barbosa Micheli, Ronaldo da Silva Dias, Michael Gaitan

**Affiliations:** 1 National Institute of Standards and Technology (NIST), Gaithersburg, MD 20899, USA; jon.geist@ieee.org; 2 National Institute of Standards (NIS), Giza 12211, Egypt; hany.amir15@gmail.com; 3 Centro Nacional de Metrología (CENAM), Querétaro 76246, Mexico; algarcia@cenam.mx (A.A.G.G.); arruiz@cenam.mx (A.R.R.); 4 Instituto Nacional de Metrologia, Normalização e Qualidade Industrial (INMETRO), Duque de Caxias 25250-020, Brazil; gbmicheli@inmetro.gov.br (G.B.M.); rsdias@inmetro.gov.br (R.d.S.D.); 5 NIST, Gaithersburg, MD 20899, USA

**Keywords:** accelerometer, accuracy, calibration, DC offset, intrinsic properties, uncertainty

## Abstract

We describe a modification of a previously described measurement–analysis protocol to determine the intrinsic properties of triaxial accelerometers by using a measurement protocol based on angular stepwise rotation in the Earth’s gravitational field. This study was conducted with MEMS triaxial accelerometers that were co-integrated in four consumer-grade wireless microsensors. The measurements were carried out on low-cost rotation tables in different laboratories in different countries to simulate the reproducibility environment encountered in inter-comparisons of calibration capabilities. We used a previously described calibration–uncertainty metric to independently characterize the overall uncertainty of the calibration and analysis process. The intrinsic property analysis suggested, and the uncertainty metric confirmed, an unacceptably large error in one combination of microsystem and low-cost rotation table. A simple modification of the analysis protocol provided a substantial improvement in the reproducibility of the protocol with all combinations of microsystem and rotation table. Later, measurements with a high-performance triaxial accelerometer using a significantly more expensive rotation table carried out at one location further validated the usefulness of this modification. The results reported here also demonstrate the existence of unidentified defects in one microsystem and one low-cost rotation table that interact with each other in ways not currently understood to produce anomalously large errors with the old protocol but not with the new protocol.

## 1. Introduction

We previously reported a measurement and analysis protocol [1,2] to determine the intrinsic properties of triaxial accelerometers. These properties include the sensitivity matrix $S_{jk}$, the intrinsic angles $\varphi_{j,j^{\prime }}$, and the DC offsets $C_{j}$, where $j,j^{\prime }\in \{u,v,w\}$ index the three accelerometers in the triaxial device with $u^{\prime }=v,v^{\prime }=w,w^{\prime }=u$, and k∈{x,y,z} index the axes of the orthonormal coordinate system to which these quantities are referenced.

In this paper, we describe the use of a calibration–uncertainty metric described in [3] to test the possibility of using a few setups consisting of a low-cost rotation stage and low-cost, wireless microsensor system to compare the triaxial–accelerometer calibration capabilities of different laboratories. During this study, we discovered that the equation used in [1] to estimate the intrinsic DC offsets introduces unnecessary calibration errors into the previously described analysis protocol. Here, we demonstrate that a different equation produces uniformly lower uncertainties in a small set of examples.

We also discovered anomalously large errors with one of the setups that we could not associate with only the DUT or only the rotation stage used in that setup. Interestingly, use of the new equation reduced the calibration–uncertainty metric of the anomalous setup below that of two of the other setups, which were also reduced by the new equation.

## 2. Theory

The calibration–accuracy metric that we used is based on the fact that a user of a calibrated triaxial accelerometer calculates the magnitude of the acceleration experienced by the accelerometer as

(1) $$a_{m}=\sqrt{a_{xm}^{2}+a_{ym}^{2}+a_{zm}^{2}},$$

where

(2) $$a_{km}=\sum_{j}T_{jk}\left(R_{jm}-C_{j}\right),$$

where $R_{jm}$ is the value of the measured output of the j accelerometer used to calculate the calibration–accuracy metric, defined as

(3) $$R_{jm}=R_{j\left(m=n+N\left(e-1\right)\right)}=R_{jne},$$

$R_{jne}$ is the response of the j accelerometer used in the intrinsic property protocol, which is defined in Equation (8), so e=1,…,3 indexes three independent rotation experiments, each having n=1,…,N rotation measurement steps in a single rotation experiment, and $T_{kj}$ is the inverse of $S_{jk}$.

Furthermore, in our original protocol,

(4) $$\left[\begin{array}{ccc} S_{jx} & S_{jy} & S_{jz} \end{array}\right]=\left[\begin{array}{ccc} A_{jx2}+A_{jx3} & {A_{jy1}+B}_{jy3} & {B_{jz1}+B}_{jz2} \end{array}\right]/{2g}_{l},$$

(5) $$A_{jke}={\pm \overset{ˇ}{A}}_{jke},$$

(6) $$B_{jk2}={\pm \overset{ˇ}{B}}_{jke},$$

(7X) $$C_{j}=(C_{j1}+C_{j2}+C_{j3})/3,$$

where ${\overset{ˇ}{A}}_{jxe}$, ${\overset{ˇ}{B}}_{jxe}$, and $C_{je}$ are adjusted in a least-squares fit to independently minimize

(8) $$R_{jne}=C_{je}+\sum_{k\in \left\{x,y,z\right\}}{\overset{ˇ}{A}}_{jke}\sin\alpha_{n}+{\overset{ˇ}{B}}_{jke}\cos\alpha_{n},$$

for each value of e, where $\alpha_{n}=\frac{2\pi \left(n-1\right)}{N},$ and $g_{l}$ is the local gravitational acceleration in the vicinity of the calibration. The signs of ${\overset{ˇ}{A}}_{jxe}$ and ${\overset{ˇ}{B}}_{jxe}$ are chosen to make the diagonal elements of $S_{jk}$ positive and the signs of the off-diagonal elements consistent with the rotation experiments [1]. The X following the 7 in Equation “(7X)” indicates that this equation should no longer be used to calculate the DC offsets in the intrinsic property analysis protocol [1], because there is a more accurate equation, as described below.

The metric in [3], which is an extension of a previously described in situ accelerometer calibration procedure [4], provides a comprehensive, type A uncertainty estimate of the calibration protocol described above. The basic idea is that a motionless, calibrated triaxial inertial accelerometer can be used to measure the gravitational acceleration in any orientation by calculating the RMS value of the three accelerometers after subtracting their offsets. Ideally, the result should be a single value equal to the gravitational acceleration $g_{l}$ for any orientation of the device.

Collecting these data for *m* orientations will result in a data series which deviation from the ideal is an indicator of the type A uncertainty in the calibrated result. After $T_{kj}$ and $C_{j}$ have been determined by measuring $R_{jm}$ for each $m=n+N\left(e-1\right)$ measurement with N=72 rotation–measurement steps, $R_{jm}$ can be substituted into Equation (2) to produce an independent estimate of each component $a_{k}$ of the local gravitational field. The results can then be substituted into Equation (1) to produce m independent estimates $g_{m}$ of the magnitude $g_{l}$ of the local gravitational field where the calibration was conducted.

The calibration–uncertainty metric is given as $V_{m}=$ $\sigma_{2}(g_{m})/g_{l}$, where $\sigma_{2}(\ldots )$ is the coverage of two standard deviations of the distribution of the $g_{m}$ data. The metric does not use the standard error, because users of the device want an estimate of the relative calibration uncertainty to assign to a single measurement to combine with other measurement–uncertainty estimates to estimate the total uncertainty in the measurement.

## 3. Materials and Method

**Figure 1:**
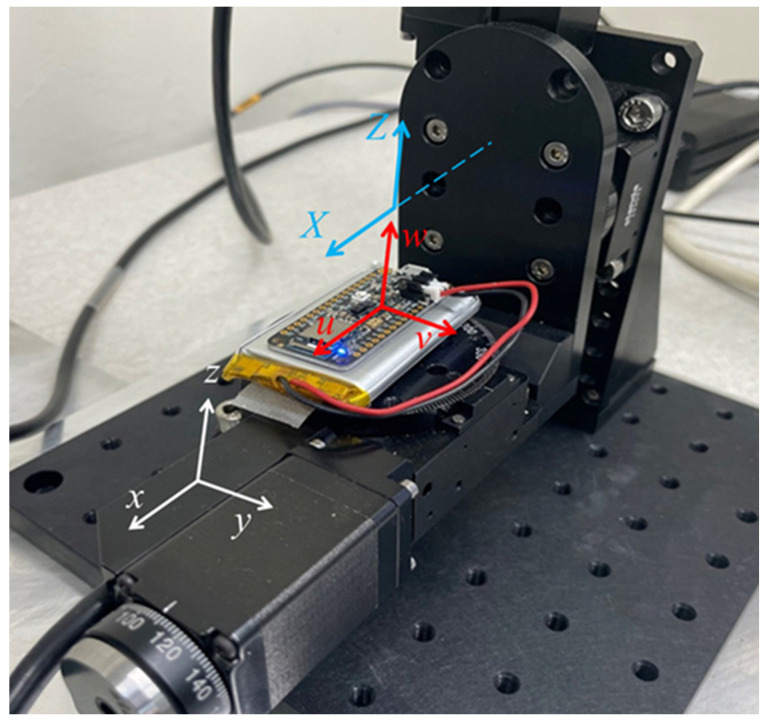
Experimental set up for measuring $T_{kj}$ and $C_{j}$ for the microsensor system shown in Figure 1. The microsensor system was glued to a battery (silver), which is mounted on the *z*-axis rotation platform of a two-axis rotation table. The local gravitational coordinate system is shown in white. The triaxial–accelerometer axes of maximum responsivity are shown in red, and the axes of rotation of the rotation table are shown in blue. A red wire connects the battery to the microsensor.

We used the calibration metric $V_{m}$ to determine the level of uncertainty available from a small sample of triaxial accelerometers (DUTs) when measured on a two-axis rotation table as an early step in designing a repeatability/reproducibility study. The two-axis rotation table shown in Figure 1 is a relatively low-cost instrument that we have been studying and comparing to high-precision rotation tables such as the one used in [1]. One type of DUT that we tested was integrated in a MEMS inertial sensor chip that was co-integrated (hybrid integration) with other MEMS sensor chips, an ADC (analog-to-digital converter) chip, a wireless-communications chip, and a microcontroller chip on a 15 cm^2^ printed circuit board to form a low-cost microsensor system.

As shown in Figure 1, each microsensor system was glued to a planar lithium battery and mounted on a two-axis rotation stage. Each microsensor system digitized the DUT output of the co-integrated triaxial accelerometer and transmitted it to the wireless transmitter system on the microsensor system. The transmitter system then passed the measurement data to a computer that collected the data wirelessly and controlled the orientation of the rotation, the rotation step size, the measurement settling time, the measurement duration at each step, and the step timing.

For the first rotation experiment (e=1), the rotation table was configured as shown in Figure 1, and a sequence of steps (wait, measure, wait, and advance 5° around rotation axis X) for n=1,…,72 was executed under computer control. For the second rotation experiment (e=2), the z−axis rotation stage was rotated through 90°, such that the v axis of the accelerometer was aligned approximately parallel to the x-axis rotation stage, and the same sequence of rotation measurement steps around the x-axis was carried out. For the third rotation experiment (e=3), starting in the configuration shown in Figure 1, the x-axis rotation stage was rotated through 90°, such that the w-axis of the accelerometer and the z-axis of the z-axis rotation stage were aligned approximately parallel to the y-axis of the local gravitational coordinate system, and the same sequence of rotation measurement steps was carried out but around the z-axis instead of the x-axis.

## 4. Results

The rotation experiments and analysis of the intrinsic properties of four DUTs were carried out following the protocol in [1] on four nominally identical DUTs (1, 2, 3, and 4) mounted on three nominally identical rotation stages (a, b, and c), as shown in Figure 1. For instance, Setup 2b would describe DUT 2 mounted on rotation stage b. Except for Setups 3c, 4c, and 5d, each rotation stage was located in a different laboratory in a different country. The results of these measurements are shown in Table 1.

## 5. Discussion

The intrinsic sensitivities

(9) $$S_{j}=\sqrt{\sum_{k\in \left\{x,y,z\right\}}S_{jk}^{2}}$$

in Table 1 are reported in units of ${1/g}_{l}$, where $g_{l}$ refers to the gravitational acceleration in the location of the measurement, to correctly compare the relative contributions of the intrinsic DC offset $C_{j}$ and sensitivity $S_{j}$ to the measured output of each accelerometer in the DUT. For instance, if $S_{u}$ for device 1 was reported in SI units, its numerical value would be 1663.5 $s^{2}m^{-1}$ to obtain a value of 16,319 when multiplied by $g_{l}=9.8101\;ms^{-2}$, the value of the local gravitational acceleration in the calibration laboratory in NIST, Gaithersburg, MD, USA. The value of $C_{u}$, however, would remain unchanged. This might give the impression that its relative contribution to the numerical value of the response of the DUT was a factor of approximately 9.8 greater than it actually is. Note, however, that units of ${1/g}_{l}$ are useful only at the location of the calibration and only for special studies where the relative value is needed, such as this study.

The relative range RR of the $S_{j}$ data is calculated as the range of the $S_{j}$ data divided by the mean of the $S_{j}$ data for each setup, and the relative range (RR) of the $C_{j}$ data is calculated as the range of the $C_{j}$ data divided by the mean of the $S_{j}$ data for each setup. Therefore, the RR values are metrics that characterize the variation of the intrinsic properties calculated from the different setups. If all the rotation tables were ideal, which is an approximation that we used in [1], then all of the variability could be attributed to the DUTs.

The values of $V_{m}$ are uncertainty metrics that characterize the error in gravitational acceleration that was reported by each DUT as its orientation was varied in a constant gravitational field. The max/min ratio of the $V_{m}$ data for the different setups is 8.3. This is a disconcertingly large ratio for data from nominally identical DUTs calibrated on nominally identical rotation tables.

Only setups 1a, 2b, and 3c were included in the original data set. To determine if DUT 3 or Rotation Table c was the source of the anomalously large errors encountered with Setup 3c, we calibrated another microsystem of the same type (DUT 4) on Rotation Table c. The value of the calibration metric $V_{m}$ for this setup was approximately one-third of its value for setup 3c, suggesting that the excess error was caused by imperfections in DUT 3. However, since the intrinsic offset $C_{w}$ for DUT 3 is greater than all of the other values of $C_{j}$, we kept this in mind when searching for an explanation for the anomalously large errors encountered with this DUT.

The values of $C_{j}$ were calculated for each accelerometer in the DUT from Equation (7X) by using all nine combinations of $j\in \left\{u,v,w\right\}$ and $e\in \left\{1,2,3\right\}$ On the other hand, each element of the sensitivity matrix $S_{jk}$ was calculated from only six of the possible nine combinations of j and e for any given value of k. In each case, the indices u1, v2, and w3 were not used. This fact, along with the fact that we were not able to find any other reason for the anomalous behavior of DUT 3, suggested that the hypothesis

(10) $$\left[\begin{array}{c} C_{u}\\ C_{v}\\ C_{w} \end{array}\right]=0.5\left[\begin{array}{c} C_{uv}+C_{uw}\\ C_{vu}+C_{vw}\\ C_{wu}+C_{wv} \end{array}\right],$$

which eliminates the diagonal element of $C_{je}$, might produce more accurate estimates of $C_{j}$ than Equation (7X). In other words, the question is whether Equation (10) will produce smaller values than Equation (7X) when used to calibrate triaxial accelerometers following the intrinsic properties’ protocols.

It turns out that this is the case, as shown in Table 2. We found that not only does the use of Equation (10) instead of Equation (7X) in Equation (2) reduce $V_{m}$ for all setups and reduce the max/min ratio from 8.2 to 2.3, but the reduction for Setup 3c gives this setup the second-best value of $V_{m}$ instead of the worst while demoting Setup 4c to the worst position, as shown in Table 2.

This result casts doubt on the preliminary conclusion that the excess error was caused entirely by DUT 3. To shed more light on this issue, we measured the intrinsic properties of a higher-performance (much more expensive) MEMS triaxial accelerometer in a wired 2.0 × 2.5 × 2.5 ${cm}^{3}$ package (DUT 5) on three different rotation tables. The first was Rotation Table c, which was used in Setups 3 and 4, the second was another Rotation Table d of the same type, and the third was a higher-performance (much more expensive) Rotation Table e. The results are shown in Table 3.

With either method (Equation (7X) or Equation (10), the higher-performance DUT and rotation table produced the lowest (best) uncertainty metric. The values of $V_{m}$ calculated with Equation (7X) are all greater than the corresponding values calculated with Equation (10), but the differences are not statistically significant at the 5% level for Setups 5e and 5d based on 216 independent measurements.

Based on these observations, it appears that the use of Equation (7X) instead of Equation (10) adds unnecessary errors under some circumstances. It also appears that defects in DUTs can interact with defects in rotation tables to produce errors that are considerably greater than the error that the same DUT would produce on an ideal rotation table and vice versa.

In retrospect, it was a mistake to include the diagonal elements of $C_{je}$ in the calculation of the offset triplet $C_{j},$ because the off-diagonal elements play a very different role than the diagonal elements. The measurement model and analysis protocol are optimized to minimize the difference between the modeled and measured response of an accelerometer that rotates around an axis oriented parallel to the gravity vector. The off-diagonal elements are quite insensitive to some phenomena that are not included in the measurement model of [1], as discussed in [2]. These include non-uniform rotation step size, hysteresis in the accelerometer response over a 360° rotation due to precession, and nutation of the rotation stage or shifts in the accelerometer DC offset signal with orientation. On the other hand, the diagonal elements of $C_{je}$ are quite sensitive to some of these phenomena. We did not understand this issue when choosing to use all of the estimates of $C_{j}$ in Equation (7X) in the original analysis protocol.

## 6. Conclusions

After reconsideration of the derivation of the analysis protocol, we concluded that that the diagonal elements of the $C_{je}$ array should not be used in the equations that defines the intrinsic offsets $C_{j}$, because the diagonal elements of the $A_{jke}$ array and $B_{jke}$ array are not used in the equations that define the sensitivity matrix $S_{jk}$. We then hypothesized that Equation (10), which does not contain the diagonal elements of the $C_{je}$ array, would produce statistically comparable or better (smaller) values of the calibration uncertainty metric $V_{m}$ than Equation (7X) for any data set produced following our intrinsic-property measurement protocol with any reasonable two-axis rotation table and any reasonable three-axis accelerometers. We then tested this hypothesis with a miniscule subset of all two-axis rotation tables and three-axis accelerometers consisting of seven combinations of two types of rotation tables and two types of accelerometers. For each combination, Equation (10) produced a comparable or smaller value of $V_{m}$ than that with Equation (7X), consistent with our theoretically derived hypothesis, thus lending support to this hypothesis. Finally, as the current “custodians” of the NIST intrinsic properties measurement and analysis protocols, we replaced Equation (7X) with Equation (10) in the protocol with the expectation that this result is quite general, even though we have confirmed it for a very small sample.

These results also show the effect of interactions of unidentified defects in a MEMS triaxial accelerometer co-integrated into a low-cost microsystem and unidentified defects in a low-cost rotation table. Furthermore, these effects cannot be described with a simple model, much less a simple linear model, even when Equation (10) is used instead of Equation (7X). Nevertheless, these results suggest that the uncertainty metric $V_{m}$ may be a useful tool for accepting or rejecting rotation tables and triaxial accelerometers for various applications, as well as characterizing the uncertainty of calibrations of triaxial accelerometers carried out by rotation in the gravitations field.

## Figures and Tables

**Table 1:** Nine intrinsic properties of the triaxial–accelerometer in four low-cost microsensor systems (1, 2, 3, and 4) of the type shown in Figure 1, measured by rotation in the gravitational field with three different low-cost two-axis rotation tables (a, b, and c) of the type shown in Figure 1. RR is the relative range of the reported values, which is not a useful concept for the intrinsic angles or the calibration–uncertainty metric $V_{m}$.

Setup	$S_{u}$	$S_{v}$	$S_{w}$	$C_{u}$	$C_{v}$	$C_{w}$	$\varphi_{u,v}$	$\varphi_{v,w}$	$\varphi_{w,u}$	$V_{m}$
	$g_{l}^{-1}$	$g_{l}^{-1}$	$g_{l}^{-1}$	none	none	none	°arc	°arc	°arc	%
1a	16,319	16,333	16,524	273	−138	421	89.98	89.88	89.77	0.214%
2b	16,338	16,406	16,422	264	−107	215	89.79	90.08	89.71	0.568%
3c	16,354	16,355	16,422	378	−5	680	90.12	90.07	89.87	1.756%
4c	16,424	16,356	16,475	14	−175	177	90.05	89.75	90.01	0.626%
RR	0.64%	0.45%	0.62%	2.23%	1.04%	3.07%	−	−	−	−

**Table 2:** Comparison of the calibration–uncertainty metric $V_{m}=$ $\sigma_{2}(g_{m})/g_{l}$ for four measurement setups when using $C_{j}$ calculated from Equation (7X) [1,2] and when calculated from Equation (10).

$C_{j}$ from\Setup	1a	3c	2b	4c	Max/Min
Equation (7X)	0.214%	1.756%	0.568%	0.626%	8.2
Equation (10)	0.191%	0.274%	0.301%	0.445%	2.3

**Table 3:** Comparison of the calibration–uncertainty metric $V_{m}=$ $\sigma_{2}(g_{m})/g_{l}$ for four measurement setups when calculated from Equation (7X) [1,2] and when calculated from Equation (10).

	$V_{m}=$ $\;\sigma_{2}(g_{m})/g_{l}$			
Method↓ \Setup→	5e	5d	5c	Max/Min
Equation (7X)	0.060%	0.072%	0.23%	3.8
Equation (10)	0.051%	0.066%	0.13%	2.5

## Data Availability

The original contributions presented in the study are included in the article, further inquiries can be directed to the corresponding author.
